# A study of thermal performance enhancement of building envelopes by using corncob as construction material

**DOI:** 10.1038/s41598-023-44206-3

**Published:** 2023-10-19

**Authors:** Yi Huang, Chong Xu, Liang Wang

**Affiliations:** 1 College of Mechanical and Electrical Engineering, Qingdao Agricultural University, Qingdao, China; 2 College of Civil Engineering & Architecture, Qingdao Agricultural University, Qingdao, China; 3 College of Architecture and Urban Planning, Qingdao University of Technology, Qingdao, China

**Keywords:** Environmental impact, Civil engineering

## Abstract

The energy-saving design of buildings effectively reduces heating energy consumption, improves indoor environmental problems in traditional houses, and plays an essential role in alleviating the increasingly severe environmental and energy problems. This study reviews the status of research on building energy-saving renovation and environmental improvement in various countries and summarizes their relevant research experiences. It described the theories and methods of survey, experiment, and numerical simulation to provide a theoretical basis for energy-saving renovation and environmental improvement of residential houses. Furthermore, it introduces corncob concrete material to provide new materials for the renovation of an old building. This research is based on the coastal Liyuan houses in Qingdao, and the problems of Liyuan houses are identified through in-field investigation. Then, through experimental simulations, the application and efficiency of corncob materials in the thermal performance improvement of the envelope are explored. The renovation of old residential houses in terms of energy-saving and environmental improvement is proposed from envelope renovation.

## Introduction

With the exploration and extension of sustainable strategy attempted in China, the economic and agricultural development level has been improved continuously. However, in the process of fast growth, the balance between human activities and natural system has been broken; thus, the pressure on the ecological environment has gradually increased. In order to fix the balance between economic growth and environmental protection, the guiding policy of "concept of Eco civilization" has gradually gained popularity. Up to now, China has gained certain achievement in the Eco civilization construction. However, it is still confronting high pressure on the ecological environment.

Agriculture, as the primary industry in China, is significant to the development of the integral national economy. Since 2017, The Green Production Policy has promoted clean production in agriculture and focused on the management of outstanding agricultural environment problems. In 2018, it was proposed to lead rural revitalization with the concept of green development. In 2019, the policy of high-quality development in agriculture promoted the improvement of rural human settlements comprehensively. In 2020, steadily improve the rural living environment was taken as one of the national development goals.

As an opportunity of further optimization, taking advantage scientifically of the agricultural by-products, which has the features of abundant yield and flexible collection, and making use efficiently of biomass resources, can reduce the pollution to the living environment and promote the adaptation process of the living environment significantly. It could eventually achieve coordinated development of life, production and ecology.

On the other hand, with the high speed in development of China's construction industry, the production of construction waste is also increasing. From preliminary statistics, the total amount of construction waste in China is about 2.1 billion to 2.8 billion tons, and the number is still increasing. The treatment of construction waste has become the major problem, which required reasonable solution urgently. With the continuous reduction of natural resources, it is especially important to improve the recycling efficiency of resources. In the outline of the 12th Five Year Plan for China's national economic and social development, it has been clearly proposed that “to accelerate the construction of a resource-saving production mode, to establish the concept of green development, and to realize improvement in Eco civilization”^1–5^.

## Materials and methods

### The research background of the material

Construction waste comes from the demolished architecture and it should provide positive benefit to it. The use of construction waste as new building material has gained more attention recently. With the acceleration of urbanization, demolition and reconstruction projects generated a large amount of construction waste, which also accelerate the shortage of new construction resources. How to make full and efficient use of construction waste, especially the trials on making recycling concrete from waste, has become a concerned topic in many countries.

The limited natural resources cannot satisfy the fast development in the construction industry gradually. The recycling concrete from construction waste can reduce the consumption of natural resources significantly. The recycling aggregate is an effective utilization of construction waste, which is economical and environmentally friendly as positive solution to the environmental problem. Under the trend of energy saving and environmental protection, the use of recycling aggregate applied in recycling concrete has become the research concerns in architecture.

The comprehensive utilization of agricultural and construction waste is one of the most economical, ecological and effective methods. The co-processing of agricultural and construction waste can turn these recycling resources into valuable treasures, effectively alleviate the shortage of resources and energy, which can reduce the pollution of solid waste to the environment, but also achieve collective long-term development in environmental, economic and social benefits.

In the process of renovation construction in China, the envelope of residential enclosures requires a large amount of energy-saving, environmentally friendly and low-cost insulation materials. Corncob is one renewable resource solution, which is convenient to obtain, energy-saving and environmentally friendly. By using corncob as concrete aggregate, the building cost could be reduced greatly. Corncob has high value as a building material, but it has not been widely used at home and abroad. Whether it can be used as an environmentally friendly building material in the construction industry still needs to be certificated in many aspects. Therefore, the application of corncob as a building material will have great potential and significant economic and social benefits possibilities^6,7^.

Therefore, this paper makes experiment on fully utilization of the features of corncob, such as light weight, heat preservation and crack resistance, etc. At the same time, it co-disposes all-component recycling sand prepared from construction waste, and jointly prepares corncob-recycled aggregate composite ecological concrete, which develops a new technical solution of green ecological building concrete materials. It provided a solution to expand the high-end utilization of agricultural and recycling construction waste products. The research conclusion of this chapter will promote the coordinated development of rural economy and society, change the mode of rural economic development, and conform to the basic national policy of environmental protection and sustainable development in my country, and conform to the green ecological concept of harmonious coexistence between man and nature.

### The situation research of material corncob

China attaches great importance to the development of circular economy, the core of which is the recycling of resources. The current ecological environment problems have attracted great attention worldwide, and the recycling of resources has important practical significance for protecting and improving the ecological environment, especially in the resource utilization of solid waste in agriculture and construction.

#### Research of agricultural solid waste-corncob

Agricultural waste is a rich source of renewable energy, but with the development of science and technology, its utilization value is getting lower and lower, and it has gradually become one of the main pollution sources of the rural environment. In order to effectively alleviate this severe situation, it is necessary to make reasonable planning for agricultural waste resources, appropriately increase the comprehensive utilization rate of agricultural waste, and realize the recycling of waste resources, so as to turn agricultural waste from "waste" into "treasure". As an important recyclable resource, corncob has not been used in high value in the process of agricultural development. It is usually treated by means of rural burning for heating, animal husbandry, and returning plow to fields, which not only pollutes the ecological environment, but also Caused a waste of natural resources^6^. In addition, corncobs are uniform in texture, light in weight, have certain strength, thermal insulation, and sound absorption performance. They are a valuable secondary resource that urgently needs to be developed and utilized.

#### Current research on resource utilization of construction waste

Construction waste comes from construction and should be returned to construction. The use of construction waste as building materials has attracted much attention. With the acceleration of urbanization, demolition or reconstruction projects will generate a large amount of construction waste, and also bring about a serious shortage of building material resources. How to fully and efficiently utilize construction waste, especially the application of waste concrete, has become a hot topic of joint research in many countries.

The limited nature of natural resources gradually does not correspond to the rapid development of the construction industry. The recycling of waste concrete can effectively reduce the consumption of natural resources. Recycled aggregate is an effective use of construction waste resources, which is both economical and environmentally friendly, and has significant benefits. Domestic research on recycled aggregates mainly focuses on the mechanical properties of recycled aggregates and recycled concrete.

Recycled aggregates have poor performance and cannot be directly used in concrete. In order to improve the utilization of recycled aggregates and promote the development of recycled concrete, it is necessary to strengthen the recycled aggregates. Li^7^ and others strengthened the quality of recycled fine aggregate, the particle size of the recycled fine aggregate after particle shaping was improved, the apparent density was slightly increased, and the water absorption rate was reduced; Guo et al.^8,9^ from the physical and the quality of recycled coarse aggregate is improved in chemical aspects. The study found that the 24 h water absorption rate of recycled coarse aggregate after secondary particle shaping treatment is reduced by 2.5%, and the crushing index is reduced by 9%. The performance parameters are close to natural aggregate; After chemical treatment of recycled coarse aggregate, its 24 h water absorption rate decreased by 2.3%, and the crushing index decreased by 5%; It also improves the performance of recycled aggregate, so as to achieve the purpose of improving the performance of recycled aggregate concrete^10–13^.

### Description of corncob materials

#### Coarse aggregate

**Figure 1:**
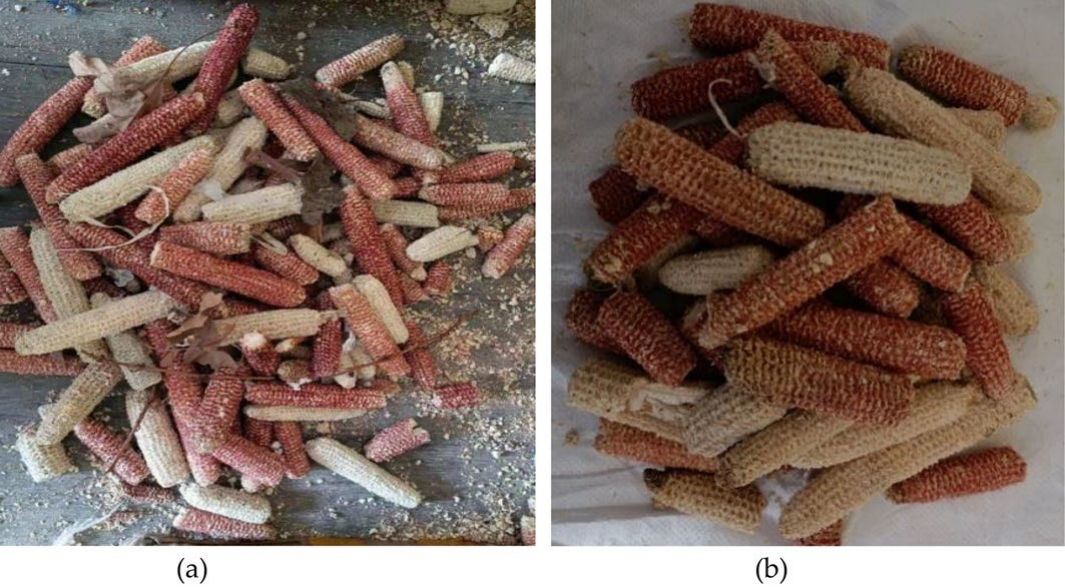
Appearance and morphology of corncob (**a**) Appearance before sorting (**b**) Appearance after sorting.

In the experimental process of the research on the performance of corncob-recycled aggregate composite ecological concrete, the selected coarse aggregate is corncob in agricultural waste. Similar to corn stover. When selecting raw materials, based on the principle of local materials, the corncob used in the experiment was provided by growers near Chengyang District, Qingdao. Considering that the corncob bone brings more impurities to the corncob during the mechanical threshing process, the corn leaves, corn silk, residual corn kernels and other magazines other than the corncob should be manually sorted and removed before the crushing treatment. The appearance and morphology of corncob before and after selection are shown in Fig. 1a and b respectively. It can be seen that many impurities attached to the surface of the corncob have been basically removed after the manual sorting treatment, which meets the requirements of the test before crushing.

**Figure 2:**
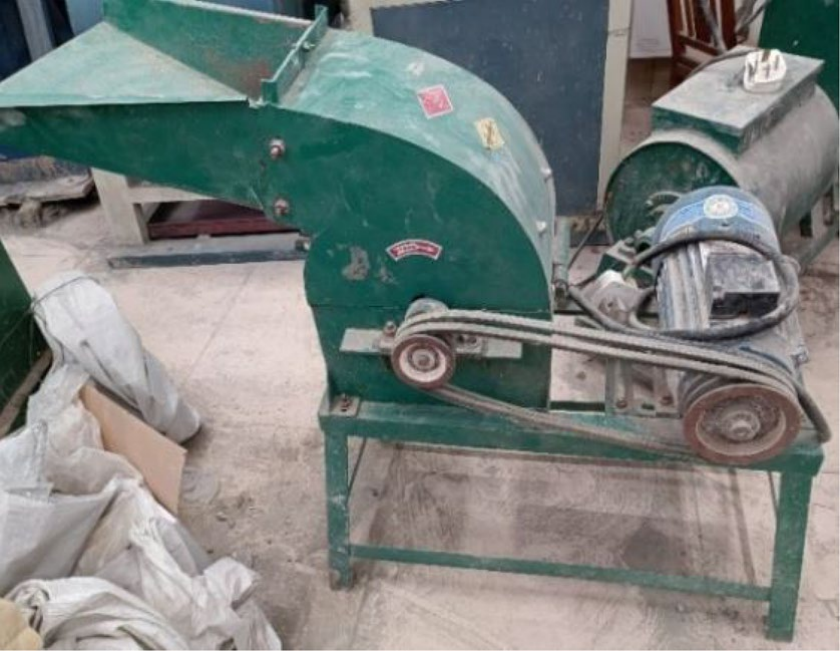
Appearance of agricultural crusher.

**Figure 3:**
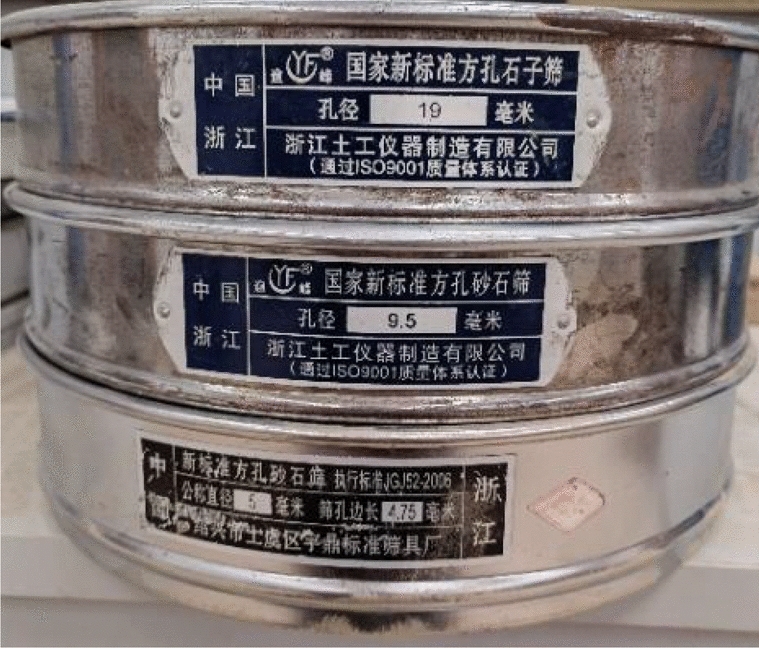
Standard square hole sieve.

In the test, the agricultural pulverizer (Fig. 2) was used for crushing treatment on the raw material of corncob after the manual sorting. In the process of corncob crushing, the diameter of the screen inside the agricultural pulverizer has a great impact on the particle size of the formed corncob particles, so it needs to be screened after the crushing process. Considering the particularity of corncob and its use as a coarse aggregate for the preparation of ecological concrete, the crushed corncob particles were sieved through square-hole sieves with side lengths of 19 mm, 9 mm and 4.75 mm in turn. The set of sieves are shown in Fig. 3.

**Figure 4:**
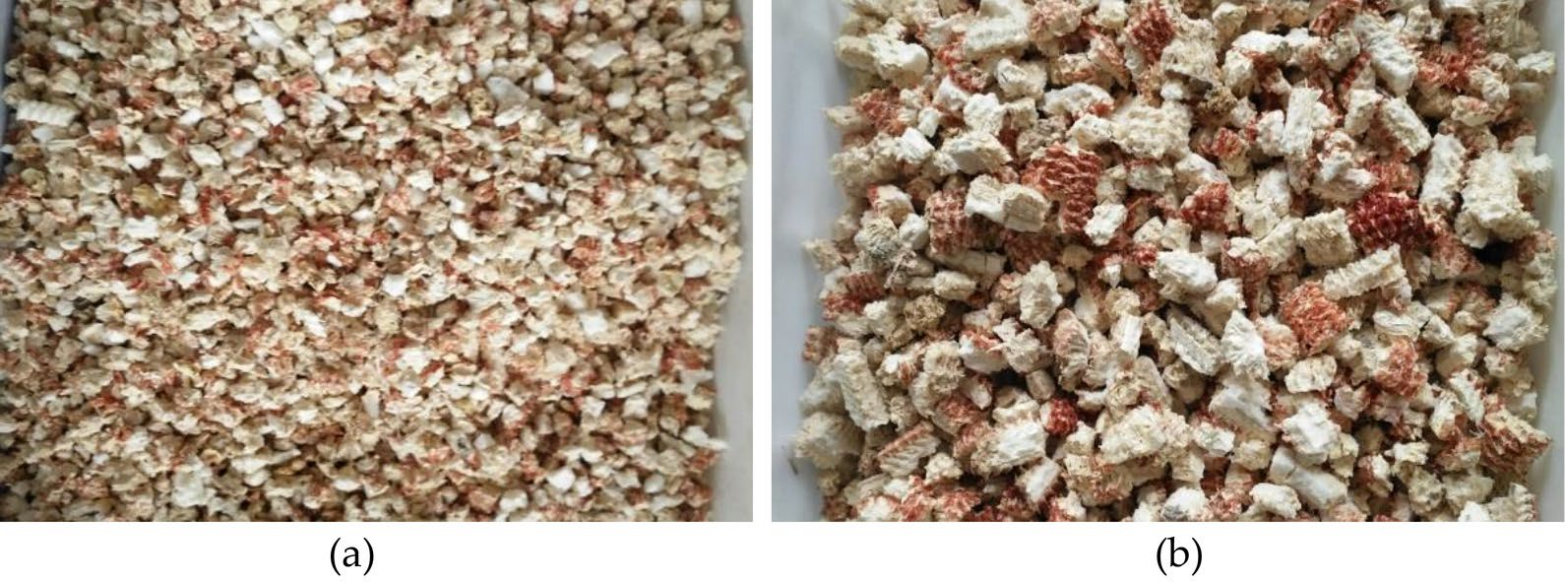
Corncob particles with different particle size ranges (**a**) 75–9 mm; (**b**) 9–19 mm.

After sieving, corncob particles with different particle sizes were obtained, and their appearance and morphology were shown in Fig. 4a and b, respectively. According to the different particle size range of corncob particles, in the experiment, the particle size range of 4.75–9 mm is set as the small-diameter corncob particle, and the particle diameter of 9–19 mm is set as the large-diameter corncob particle. According to the test needs and bulk density requirements, the mixing ratio of small-sized corncob particles and large-sized corncob particles is determined to be 1:1.5, and the mixed corncob particles are used for preparing corncob-recycled aggregate composite ecological concrete of coarse aggregate.

#### Fine aggregate

When the service life of some buildings is too long, the performance of recycled coarse aggregate is weakened, and the dismantled construction waste is no longer suitable for use as coarse aggregate for recycling; or the building itself cannot continue to build due to the quality of coarse aggregate. When the construction waste is completely crushed into fine aggregate due to construction requirements, the construction waste can be completely crushed into fine aggregate, which is called full-component recycled sand, which is the fine aggregate used in the test.

In the preparation process, the preparation process of full-component reclaimed sand can be subdivided into full-component simple crushing reclaimed sand and full-component particle shaping reclaimed sand. The main feature of the whole-component particle shaping reclaimed sand preparation process is that all the waste concrete blocks are used to prepare the reclaimed sand. That is, during the initial simple crushing, the natural coarse aggregate in the original waste concrete is no longer used to prepare the recycled coarse aggregate, but is all crushed into recycled sand. increase accordingly.

However, the performance of full-component particle shaping regenerated sand is better than that of full-component simple crushing regenerated sand, and the economic cost increases less. Therefore, in the experiment, the regenerated sand with full-component particle shaping was finally selected as the fine aggregate for preparing corncob-recycled aggregate ecological concrete.

### Preparation methods of aggregate

Coarse aggregate corncob is obtained by sorting, crushing and screening of corncob bones, and the waste concrete is crushed and reshaped to obtain full-component regenerated sand, which is used as the fine aggregate for this test; The water consumption, the two together constitute the water consumption of the concrete test, and the mixing ratio plan is designed with the mortar ratio and the volume content of the corncob as the influencing factors.

By exploring the relationship between the mass and volume of the corncob, the actual mixing quality of the corncob was determined; the basic performance (dry density and Thermal conductivity) and mechanical properties (cube compressive strength under different curing age conditions), and systematically explore the change law of its performance by analyzing the measured test results.

Based on the experimental research, using the method of combining data analysis and theoretical analysis, the co-processing of agricultural waste and construction waste is used to prepare corncob-recycled aggregate composite ecological concrete with significant environmental protection effect. The performance provides a certain theoretical basis for the later application and promotion of agricultural engineering.

### Preparation methods of composite ecological concrete with corncob-recycled

**Figure 5:**
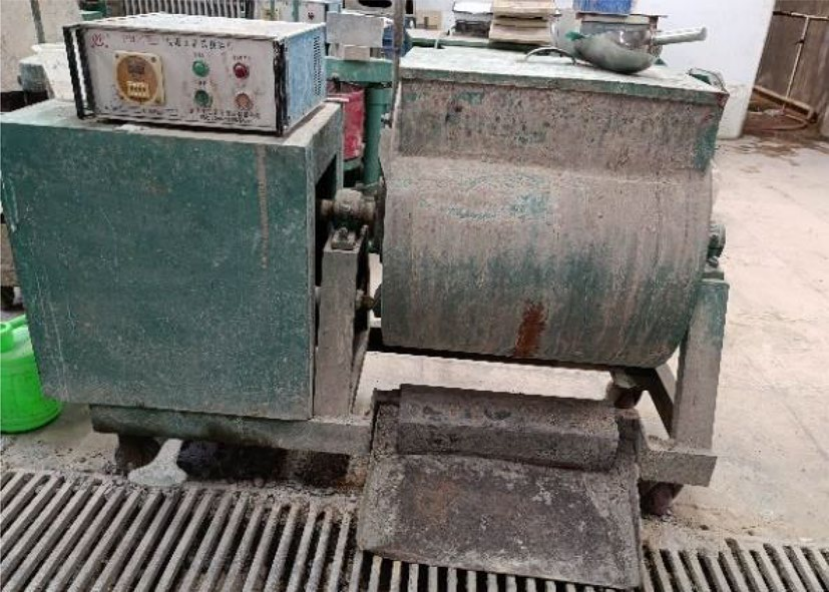
Concrete horizontal mixer.

In the test, a horizontal mixer was used to prepare a corncob-recycled aggregate composite ecological concrete mixture, as shown in Fig. 5. The specific feeding sequence is:

1. Add reclaimed sand and cement and mix well.
2. Add water and water reducing agent for stirring, and the stirring time is controlled within the range of 10–15 s.
3. Add the mixed corncob particles, and after stirring for 2–3 min, put the concrete mixture into the test mold.
4. Use a vibrating table to vibrate, compact and smooth.

**Figure 6:**
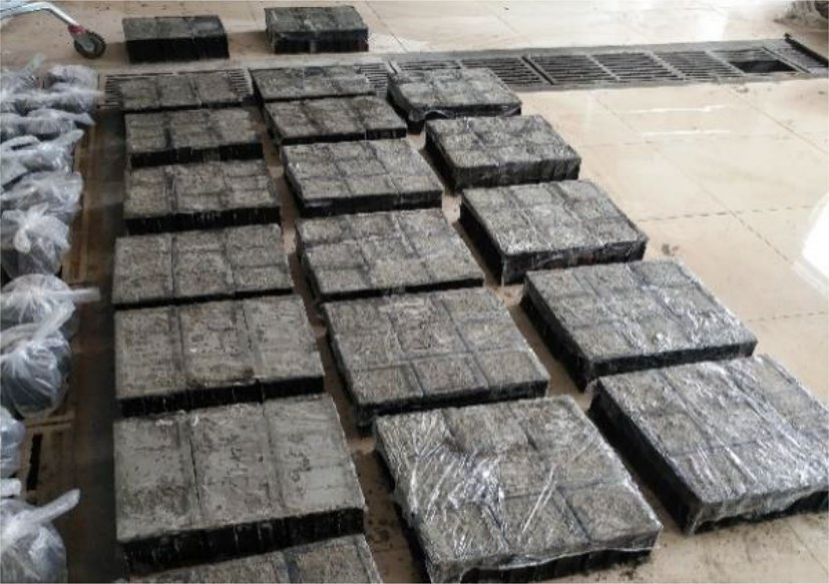
Concrete with formwork maintenance.

Because the corncob is light in texture, it will float up during the vibration process, so the vibration time should not be too long, and it is appropriate to control it within 3-5 s in the test. In addition, after the ecological concrete is installed, in order to prevent its moisture from evaporating, it should be immediately covered with plastic wrap to lock the moisture, and marked for easy identification, as shown in Fig. 6.

### Ethics approval

All the authors declared the collection and use of corncob materials applied in this study complied with relevant local institutional, national and international standards and legislation.

## Results

This section may be divided by subheadings. It should provide a concise and precise

### Preparation methods of aggregate

The specific preparation process of the full-component particle shaping regenerated sand is as follows: firstly, the waste concrete blocks are simply crushed into 6–7 mm particles by a jaw or hammer crusher, and then they are shaped by a particle shaping machine. Sand is called full-component particle shaping regenerated sand. Because the waste concrete contains about 50% of natural coarse aggregate, the full-component particle shaping reclaimed sand contains a large amount of reclaimed sand generated from natural coarse aggregate, which can effectively improve the firmness of the reclaimed sand. According to the relevant requirements in GB/T25176-2010 “Recycled Fine Aggregates for Concrete and Mortar”, the main performance indicators of the regenerated sand with full-component particle shaping are tested.

1. Particle gradation
  Comparing the regenerated sand with full-component particle shaping and natural sand prepared in the test, the particle gradation and fineness modulus are shown in Table 1. It can be seen that the regenerated sand used has a good gradation and is medium sand in Zone II.

**Table 1:** Comparison of particle gradation between full-component particle-shaping regenerated sand and natural sand.

The types of sand		Full-component particle-shaping regenerated sand	Natural sand
Mesh size (mm)	4.75	0.3	0.0
	2.36	10.3	4.8
	1.18	27.0	14.8
	0.06	48.1	32.0
	0.03	70.4	69.7
	0.15	86.2	97.8
	Weight of screen residue	100	100
	Fineness modulus	2.4	2.2
2. Mud content (Micro powder content)
  Generally, reclaimed sand is generated by crushing natural stones. The particles with a particle size of less than 75 μm in the sand are stone powder, while the particles with a particle size of less than 75 μm in the test concrete regenerated sand are mainly composed of cement stone powder, stone powder and soil. Therefore, the concrete is recycled. The content of particles with a particle size of less than 75 μm in the sand is defined as the content of fine powder. Table 2 shows the comparison of the mud content (micro powder content) between the regenerated sand with full-component particle shaping and the natural sand. It can be seen that the micro powder content of the full-component particle-shaping regenerated sand is higher than that of the natural sand, which is mainly related to the fact that the full-component particle-shaping regenerated sand contains a large amount of cement stone powder.

**Table 2:** Comparison of mud content/fine powder content between full-component particle-shaping regenerated sand and natural sand.

The types of sand	Full-component particle-shaping regenerated sand	Natural sand
Mud content/ Micro powder content (%)	4.00	0.95
3. Mud contenty
  The mud content of the reclaimed sand can be understood as the cement stone chips attached to the surface of the reclaimed sand, washed with water, and scattered by hand. The specific test results are shown in Table 3. The test results show that the full-component particle shaped regenerated sand is relatively completely broken during the preparation process, and most of the cement stone chips attached to the natural sand or coarse aggregate in the original waste concrete are ground away and exist in the form of micro powder in the whole group. The regenerated sand is shaped into particles so that the mud content is close to that of natural sand.

**Table 3:** Comparison of mud lump content between full-component particle-shaping regenerated sand and natural sand.

The types of sand	Full-component particle-shaping regenerated sand	Natural sand
Mud content (%)	2.2	1.4
4. Solidity
  In this test, the single-stage maximum crushing index is used to test the firmness of the reclaimed concrete. The larger the single-stage maximum crushing index is, the lower the firmness is. The specific test results are shown in Table 4. The results show that the full-component particle shaping reduces the single-stage maximum crushing index of the reclaimed sand to 14.0%, which is only 1.2% higher than that of the natural sand.

**Table 4:** Comparison of firmness of regenerated sand with full-component particle shaping and natural sand.

The types of sand	Full-component particle-shaping regenerated sand	Natural sand
Solidity (%)	14.0	12.8
5. Density
  The density of sand includes bulk density, compact density, apparent density and porosity. The specific test results are shown in Table 5. The results show that the bulk density, compact density and apparent density of the regenerated sand with full-component particle shaping are on average 40 kg/m^3^ lower than those of the natural river sand and are close to the natural sand in terms of density performance.

**Table 5:** Comparison of density and porosity between full-component particle-shaping regenerated sand and natural sand.

The types of sand	Full-component particle-shaping regenerated sand	Natural sand
Bulk density (kg/m^3^)	1382	1454
Compact density (kg/m^3^)	1587	1555
Apparent density (kg/m^3^)	2518	2597
Porosity	37	40
6. Water demand ratio and strength ratio of reclaimed rubber sand
  In the test, reclaimed sand and standard sand were used to prepare reclaimed sand and standard sand that meet the fluidity requirements, respectively. The water demand ratio of reclaimed sand and the strength ratio of reclaimed sand measured in the experiment are shown in Table 6. The situation is shown in Fig. 7a–c respectively.

**Table 6:** Water requirement ratio and strength ratio of full-component particle shaping regenerated sand.

The types of sand	The water demand ratio	Strength ratio
Full-component particle-shaping regenerated sand	1.27	0.87

**Figure 7:**
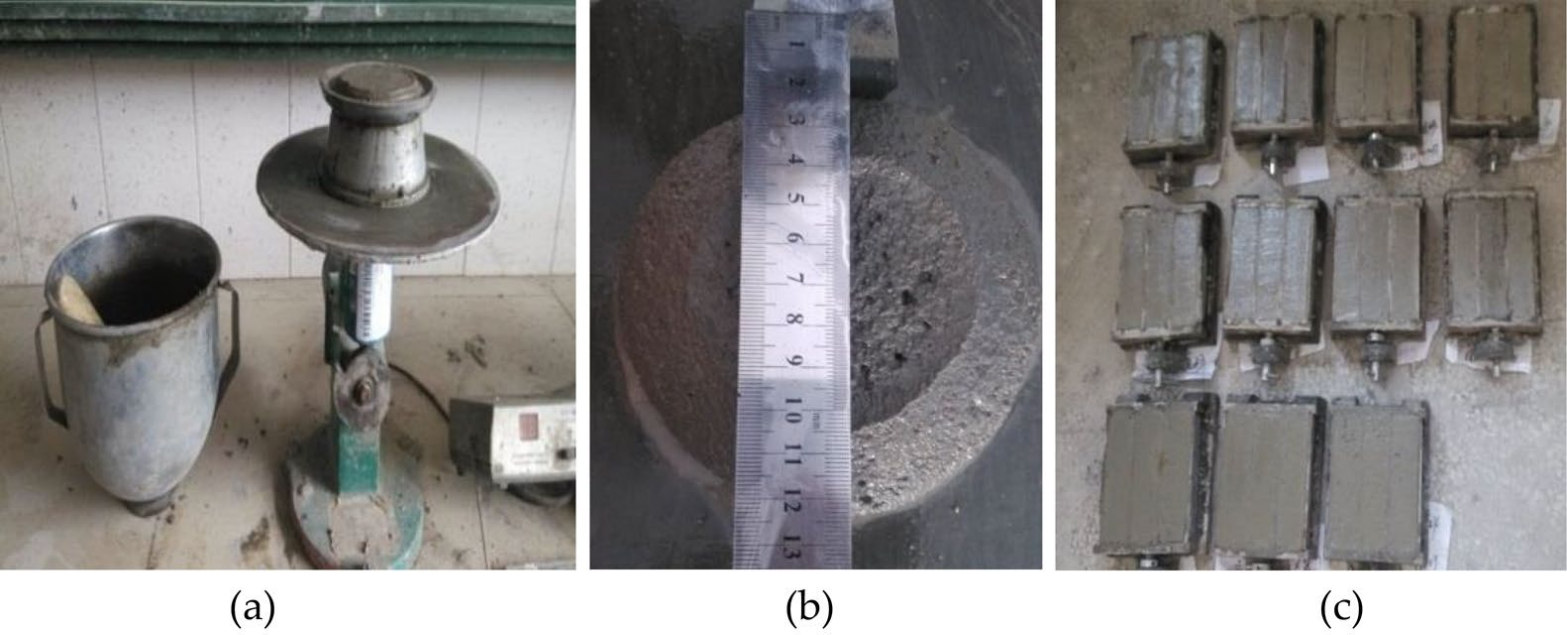
Test situation of reclaimed mortar (**a**) Preparation of reclaimed mortar (**b**) Measurement of fluidity of reclaimed mortar (**c**) Molding of reclaimed mortar.
7. Cementitious materials
  The fast-hardening sulfo-aluminate cement SAC42.5 is selected. The aliphatic superplasticizer produced by a building material company in Qingdao is used. Using ordinary tap water, in line with JGJ63-2006.

## Findings and discussion

### Basic performance properties from experiments

#### Experimental mixing proportion design

1. Determination of corncob quality
  Add 50 g, 60 g, 70 g, 80 g, 90 g, and 100 g of mixed corncob particles to the standard mortar test (mortar ratio 1:3, water-binder ratio 0.5), stir evenly, and put them into a regular container, and then observe the change in the height of the mortar surface.
  With the change in the number of mixed corncob particles, by actually measuring the height of the freshly mixed mortar in the regular container, the linear relationship between the mass and volume of the corncob is obtained. Based on the linear relationship of corncob mass-volume, the ratio of corncob to the total volume can be calculated, and then the ratio of corncob to cementitious material can be determined according to the different volume contents of corncob set in the experiment (defined as cob glue ratio), and finally, the quality of corncob used for preparing corncob-recycled aggregate composite ecological concrete can be calculated.
2. Determination of test water consumption
  The water consumption of the test should consider the water consumption of the mortar and the water consumption of the corncob, that is, the total water consumption = the water consumption of the mortar + the water consumption of the corncob.
3. Determination of water consumption for mortar
  In order to ensure that the concrete mixture has good working performance, the fluidity of the mortar is controlled to be 175–180 mm, and the amount of aliphatic water reducing agent is 2% of the amount of the cementitious material. The results are shown in Table 7.

**Table 7:** Determination of water consumption for mortar.

Cement sand ratio	Cement (kg/m^3^)	Sand (kg/ m^3^)	Water-reducing admixture(kg/ m^3^)	The amount of water (L)	Water to binder ratio
1:3	400	1200	8	174	0.434
1:3.5	400	1400	8	207	0.517
1:4	400	1600	8	227	0.567
4. Determination of corncob water consumption
  Due to the different harvesting time and storage environment of corncob, the water content of corncob itself is quite different. The water absorption test of corncob used in this experiment should be carried out in advance. During the test, take a certain mass of dry-weight mixed corncob particles and soak them in water, and measure the quality of the corncob at intervals of 10 min until the weight no longer increases, and record the quality of the corncob at this time. The water absorption rate of corncob is calculated according to formula (1):
  1 $$\omega =\frac{M1-M}{M}\times 100\%$$
  In here: ω—Corncob water absorption/%; M1—The quality of corncob after water saturation/g, M—The mass of corncob in dry weight state/g;

After calculation, the arithmetic mean of the three test results was taken as the water absorption rate of the corncob used in this test. Finally, the water absorption rate of the test corncob was measured to be 120%.

**Figure 8:**
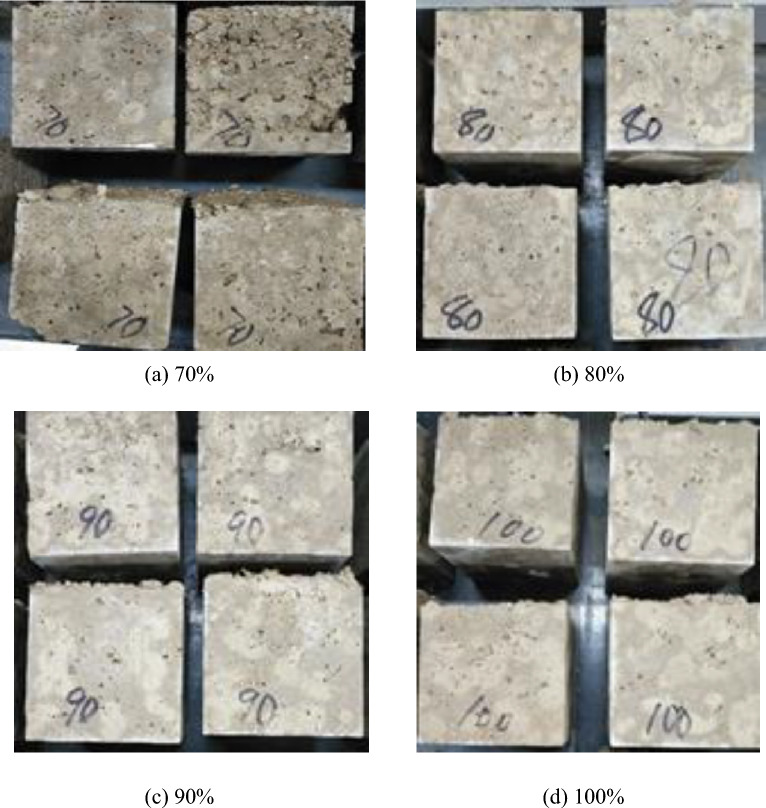
Concrete specimens prepared with different actual water consumption of corncob.

The porous organic fiber structure of corncob makes it highly absorbent, and too much moisture will not only reduce the strength of concrete, but also easily cause the concrete to crack later. In this experiment, fast-hardening sulfoaluminate cement was selected, which has a fast-setting speed, and the corncob takes a short time to absorb free water in the mixture, and it cannot reach water saturation in a short time, resulting in concrete bleeding. Taking the cement-sand ratio of 1:3 and the corncob volume content of 40% as an example, the experiment was carried out. First, the actual water consumption of the corncob was controlled to be 70%, 80%, 90% and 100% of the water absorption of the corncob, and the corncob was prepared in turn. The core-recycled aggregate composite ecological concrete cube compressive test block is used to optimize the optimal water demand of corncob by compressive strength. The test situation and results are shown in Fig. 8.

#### Thermal conductivity change

In the test, the influence of two variable factors on the performance of corncob-recycled aggregate composite ecological concrete was mainly considered. The specific changes are as follows:

1. Mortar ratio: take 1:3, 1:3.5 and 1:4 respectively.
2. Corncob volume content: take 30%, 35%, 40%, 45% and 50% respectively.

Yang Xiufei et al.conducted a repeatable experiment on thermal conductivity of 6 biomass materials in rural areas, including daylily stalk, sawdust, rice straw, rice husk, peanut husk and corn stalk. The thermal conductivity of biomass materials is between 0.0504 W/(m K) and 0.0790 W/(m K), and the thermal insulation is good, which provides a feasibility and theoretical basis for biomass materials as thermal insulation building materials. Generally speaking, the thermal insulation properties of materials mainly depend on the thermal resistance. The lower the thermal conductivity of building materials, the better the thermal insulation effect, and the thermal conductivity of different biomass materials varies^14,15^. Corncob is a biomass resource with low thermal conductivity. As a building material, it can improve the thermal insulation performance of ecological concrete. The test results of thermal conductivity of corncob-recycled aggregate ecological concrete are shown in Table 8.

**Table 8:** Test results of thermal conductivity of corncob-recycled aggregate composite ecological concrete.

Mortar ratio	Water reducing agent/kg/m^3^	Corncob volume content/%	Water consumption/L	Coefficient of thermal conductivity W/(m K)
1:3	6.4	30	210.9	0.2479
1:3	6.0	35	208.5	0.2392
1:3	5.6	40	205.5	0.2289
1:3	5.2	45	200.2	0.2177
1:3	4.8	50	193.5	0.2011
1:3.5	5.8	30	222.3	0.2421
1:3.5	5.4	35	215.6	0.2323
1:3.5	5.0	40	207.6	0.2184
1:3.5	4.6	45	198.4	0.2131
1:3.5	4.5	50	199.5	0.1857
1:4	5.2	30	220.7	0.2403
1:4	4.8	35	214.4	0.2151
1:4	4.6	40	215.6	0.2120
1:4	4.5	45	218.5	0.2109
1:4	4.3	50	218.3	0.1803

### Influence of corncob volume content on compressive strength of biomass concrete

**Figure 9:**
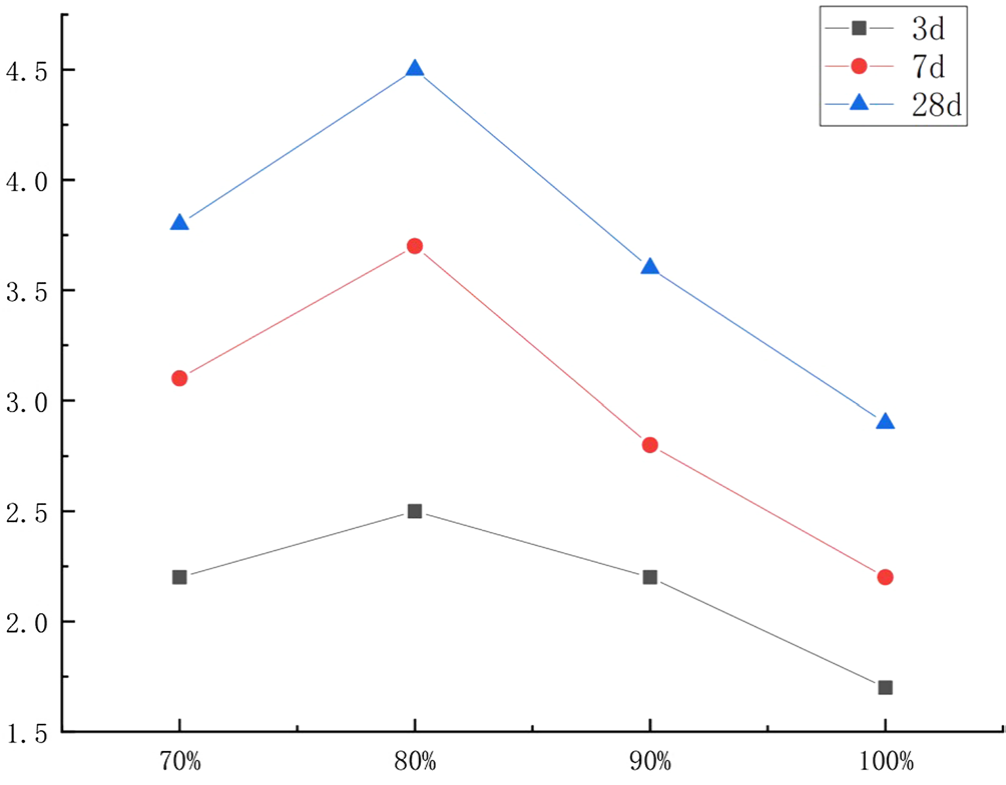
Compressive strength of concrete under different water consumption of corncob.

The test results showed that the dry density of concrete blocks gradually decreased with the increase of corncob stalks. With the more increase of the amount of corn stalks, the further decrease of concrete compressive strength. There is an obvious linear relationship between the amount of corn stalks added and the compressive strength of concrete (Fig. 9). Compared with sand and gravel resources, biomass resources have the characteristics of light weight, many pores, and high-water absorption. When used as building materials, they might reduce the dry density and compressive strength of concrete obviously. The test results proved that the higher the replacement rate of recycled fine aggregate, the more water consumption of recycled concrete mixture, and the lower the compressive strength.

### Influence of corncob volume content on thermal conductivity of ecological concrete

**Figure 10:**
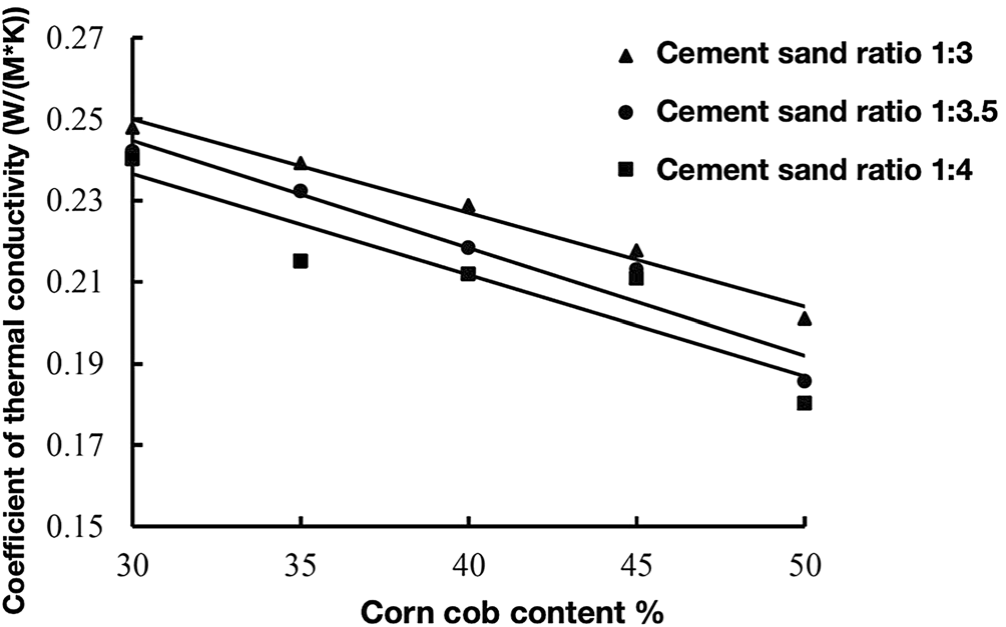
Corncob volume content-thermal conductivity curve.

Corn stalks and corncob are by-products of the same plant. At present, the use of corn stalks as thermal insulation building materials has been studied, but corncob has not been widely used. In engineering, materials with thermal conductivity less than 0.23 W/(m K) are called thermal insulation materials. The thermal conductivity of corncob is less than 0.23 W/(m K), so it can be used in buildings to reduce the thermal conductivity of concrete, thereby improving Thermal insulation properties of concrete. Therefore, in this experiment, the influence law of the thermal conductivity of corncob-recycled aggregate ecological concrete was studied, and the results are shown in Fig. 10.

As can be seen from Fig. 10, under the conditions of different cement-sand ratios, with the increase of the volume content of corncob, the thermal conductivity of ecological concrete gradually decreases. This is because the thermal conductivity of ecological concrete depends on the pore structure of the concrete itself and the thermal conductivity of the air inside the concrete. Among common substances, air has the lowest thermal conductivity. When the volume content of the corncob is large, the independent pores in the ecological concrete structure increase, and the air content inside the concrete increases, so the thermal conductivity of the ecological concrete decreases, which can achieve the effects of heat insulation and heat loss prevention.

In addition, when the ratio of mortar to sand is 1:3, the thermal conductivity of eco-concrete is less discrete. When the volume content of corncob is 30%, the thermal insulation of ecological concrete is relatively weak, and the thermal conductivity of concrete is about 0.2479 W/(m·K). When the volume content of corncob increases to 50%, the thermal conductivity of ecological concrete decreases. It is 0.2011 W/(m K), that is, for every 5% increase in the volume content of corncob, the thermal conductivity decreases by about 4.7%, and the decrease is relatively large. It can be seen that the corncob aggregate has a significant effect on the thermal conductivity of ecological concrete.

### Influence of cement-sand ratio on thermal conductivity of ecological concrete

**Figure 11:**
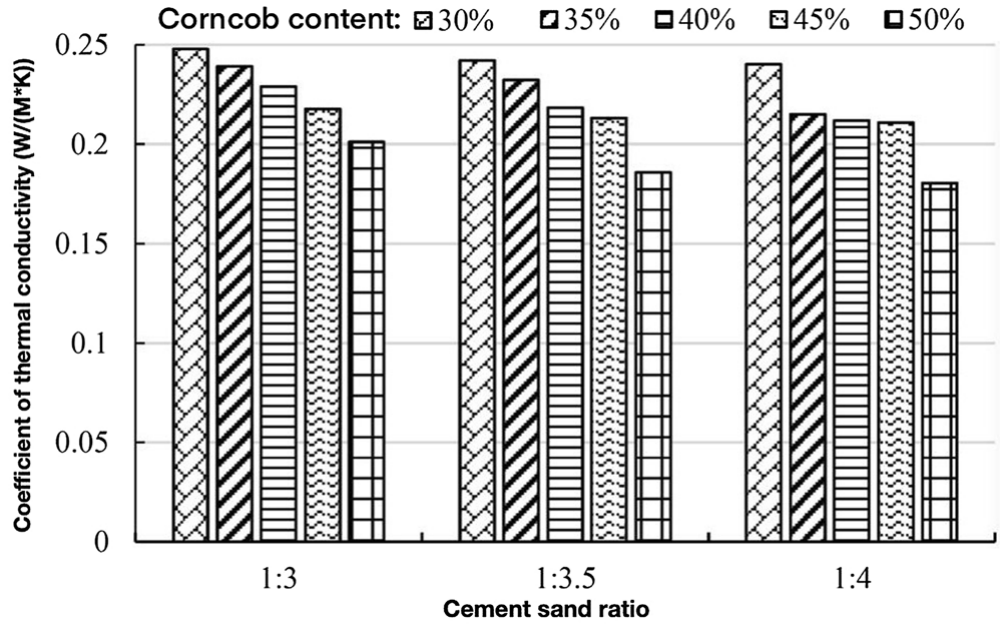
The relationship between the cement sand ratio and the thermal conductivity.

The thermal conductivity of concrete is inseparable from the constituent materials. In addition to recycled coarse aggregates, the influence of cementitious materials and recycled fine aggregates on the thermal conductivity of concrete cannot be ignored. The effect of different cement-sand ratios on the thermal conductivity of ecological concrete is shown in Fig. 11.

The recycled fine aggregate produces cracks during the crushing process, which increases the internal porosity of the concrete, increases the water absorption rate, and increases the air content, resulting in a decrease in the thermal conductivity of the corncob-recycled aggregate ecological concrete.

As can be seen from Fig. 11, with the increase of the proportion of recycled sand, the thermal conductivity of ecological concrete gradually decreases, but the highest is not more than 0.25 W/(m K), and the thermal insulation performance is better than that of ordinary concrete. On the other hand, when the volume content of corncob is 30% and the ratio of mortar to sand is 1:3, the thermal conductivity of ecological concrete is 0.2479 W/(m K). The thermal conductivity of ecological concrete with a ratio of 1:4 is the best, and its thermal conductivity is 0.2403 W/(m K). When the content of recycled sand increases by 5%, the thermal conductivity of ecological concrete decreases by about 3.1%, with a small decrease. The thermal conductivity of ecological concrete when the ratio of mortar to sand is 1:3.5 is close to the thermal conductivity of ecological concrete when the ratio of mortar to sand is 1:4. It can be seen that the effect of recycled fine aggregate on the thermal conductivity of ecological concrete is smaller than that of corncob on the thermal conductivity of ecological concrete.

In general, the thermal conductivity of corncob-recycled aggregate composite ecological concrete is between 0.1803 W/(m K) amd 0.2479 W/(m K) under the condition of different mortar ratio and corncob volume content, which is very close to the thermal insulation material, and provides a basis for the research on the preparation of energy-saving thermal insulation wall panels.

## Conclusions

This section systematically studies the influence of different cement-sand ratios and corncob volume content on the thermal conductivity of corncob-recycled aggregate composite ecological concrete, and draws the following conclusions:

1. The corncob itself has high porosity, loose pores, and high-water absorption. With the increase of the volume content of the corncob, the water absorbed by the ecological concrete increases. In the dry state, the dry density of the ecological concrete varies with the ratio of mortar to sand. It gradually decreased with the increase of corncob volume content.
2. Corncob is a lightweight, loose and porous fibrous material that retains a large amount of air in the pores of the corncob and is a biomass material with a small thermal conductivity. As the volume content of the corncob increases, the independent pores in the ecological concrete structure increase, and the air content inside the concrete increases, the thermal conductivity of the ecological concrete decreases, and the thermal insulation performance is better, which can achieve the effects of heat insulation and heat loss prevention. In the end, the corncob concrete material with the cement-sand ratio of 1:4, the water consumption of 218.3 kg/m^3^ and the volume content of corncob of 50% was selected, and its thermal conductivity was 0.18.
3. The texture of corncob is uniform, but it is lightweight and porous, and its own compressive strength is low. With the increase of the volume content of corncob, the compressive strength of ecological concrete shows a downward trend; with the continuous reduction of the mortar ratio, the proportion of recycled sand increases, the content of cementitious materials in ecological concrete is reduced, the bonding force between aggregates is weakened, and the porosity is further increased due to insufficient aggregate wrapping, which makes the compressive strength of ecological concrete continue to decrease. Under the conditions of different mortar ratio and volume content, the 28-day compressive strength of ecological concrete is between 2.1 and 5.4 MPa. Although it is lower than the compressive strength of ordinary concrete, it can still meet the requirements of masonry.

## Data Availability

The datasets during and analyzed during the current study are available from the corresponding author on reasonable request.
